# Supplementary material for: Long-range, non-local switching of spin textures in a frustrated antiferromagnet
Source: Nat Commun. 2023 Aug 4;14:4691. doi: 10.1038/s41467-023-39883-7 (PMC10403493; doi:10.1038/s41467-023-39883-7)
Supplement: Supplementary file 1 — Supplemental Information [file 41467_2023_39883_MOESM1_ESM.pdf]

SUPPLEMENTARY INFORMATION: Long-range, Non-local Switching of Spin Textures in a Frustrated Antiferromagnet

Shannon C. Haley *,[1,2] Eran Maniv,[3] Shan Wu,[1] Tessa Cookmeyer,[1,2]
Susana Torres-Londono,[1] Meera Aravinth,[1] Nikola Maksimovic,[1,2]
Joel Moore,[1,2] Robert J. Birgeneau,[1] and James G. Analytis *[1,2,4]

[1]*Department of Physics, University of California, Berkeley, CA 94720, USA*

[2]*Materials Sciences Division, Lawrence Berkeley National Laboratory, Berkeley, California, 94720, USA*

[3]*Department of Physics, Ben-Gurion University of the Negev, Beer-Sheva 84105, Israel*

[4]*CIFAR Quantum Materials, CIFAR, Toronto, Ontario M5G 1M1, Canada*

(Dated: June 28, 2023)

Strain

Uniaxial strain measurements were performed by mounting a device on a picma chip actuator. The device was about 3.5μm thick with pulse bars about 5μm wide, and was mounted on Stycast. All measurements were performed at 2K. From top to bottom in Fig. S1, the switching was conducted on the device with: no voltage (strain) applied, 40V applied at 2K, 40V applied during cooldown, and no voltage applied following the previous measurements. No difference is seen when strain is applied solely at 2K, but a sign flip is observed at about $50kA/\text{cm}^2$ when the device is cooled with 40V applied to the piezo, highlighted in blue. The amplitude of the switching is also slightly suppressed for that preparation.

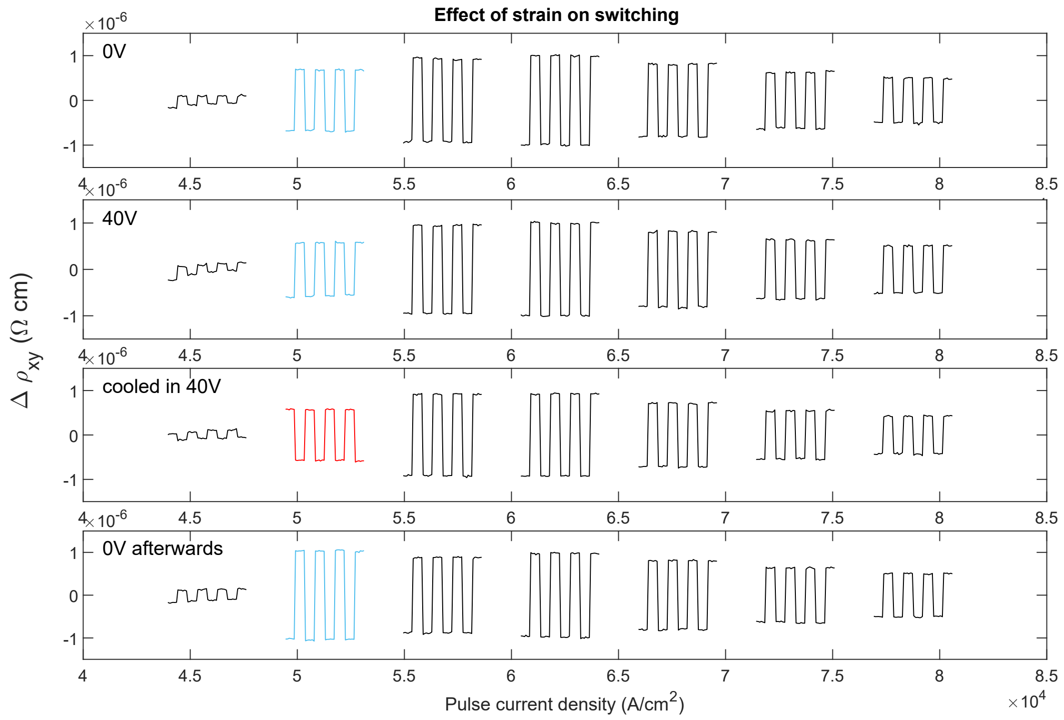

FIG. S1. Local switching as a function of pulse current density measured for a device with and without applied strain.

Non-local measurements in $Fe_{0.33}NbS_2$

Non-local measurements were performed on a sample of $Fe_{0.33}NbS_2$. While a weak reversible switching signal is observed in the local channel, the nonlocal channel sees jumps

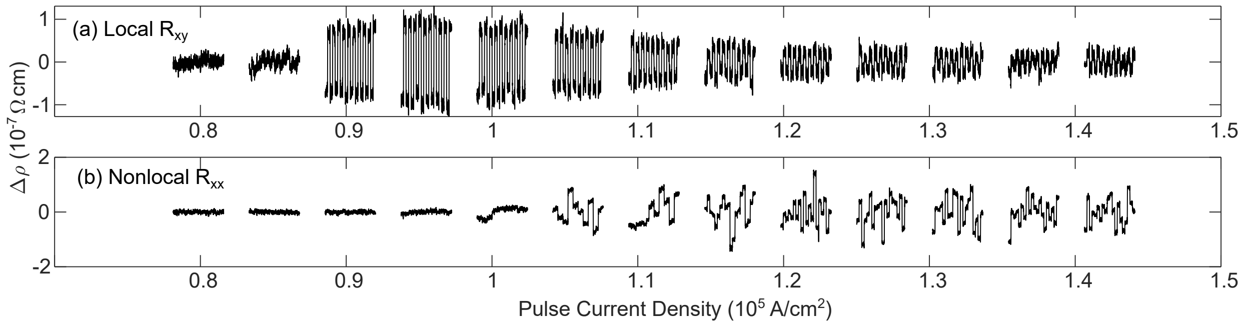

FIG. S2. (a) Local switching measurement as a function of pulse current density observed with a device of $Fe_{0.33}NbS_2$. (b) Non-local longitudinal resistivity changes observed in the same device during the same switching events.

that do not move back and forth between stable resistance states.

PXRD

High-resolution synchrotron powder x-ray diffraction measurements were taken on samples of $Fe_{0.31}NbS_2$ and $Fe_{0.35}NbS_2$ at room and low temperature, as shown in Figs. S3, S4, S5, and S6. Rietveld refinements were performed to determine the lattice paramters in each of these cases.

Full temperature dependence

Current density dependence was measured every $5K$ between 5 and $40K$ for local and non-local contacts. These pulse trains are shown in Figs.S7, S8, and S9.

Switching with lead detachment

Measurements were performed wherein the AC probe current was turned off and its leads were detached during the switch events. The leads were reattached and the probe current turned back on in order to measure the resistance between switching events. This is shown in Figs. S10 and S11. The non-local switching behavior persists, and its character does not change from that seen when the AC probe current is always on.

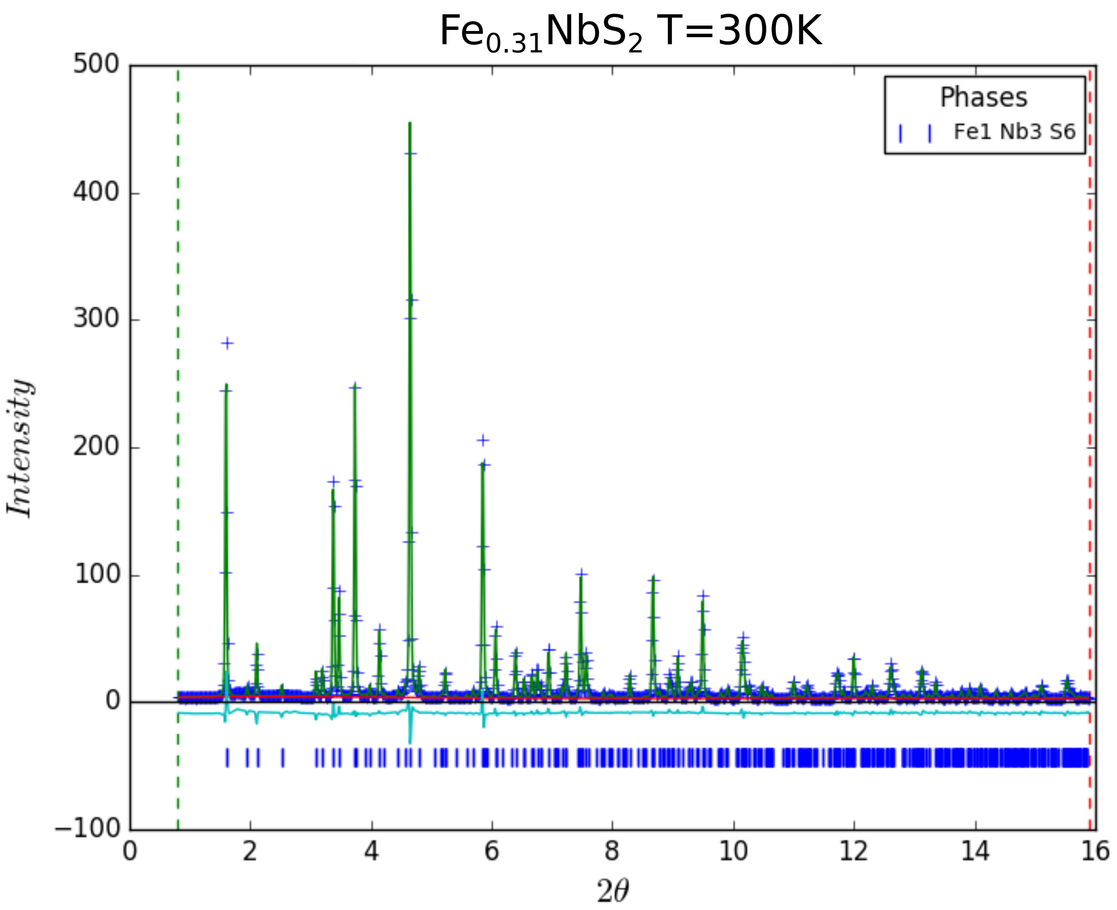

FIG. S3. Rietveld refinement of high-resolution synchrotron powder XRD measurements on $Fe_{0.31}NbS_2$ at $300K$. Calculated lattice parameters are a = 5.662078 +/- 0.000137 Å, c = 11.964578 +/- 0.000302 Å. The cross markers are data with the fit shown by the green curve, and the difference between the fit and data is shown in cyan. Vertical lines denote structural peak positions.

Probe current frequency dependence

The non-local switching behavior has no discernible dependence on the frequency of the AC probe current. See Fig. S12.

Schematic

A schematic view of the proposed mechanism for resistance switching is shown in Fig.S13. Following a horizontal current pulse in a stripe-dominated sample, the domain with principal axis parallel to the current pulse is disfavored, resulting in a combination of the other

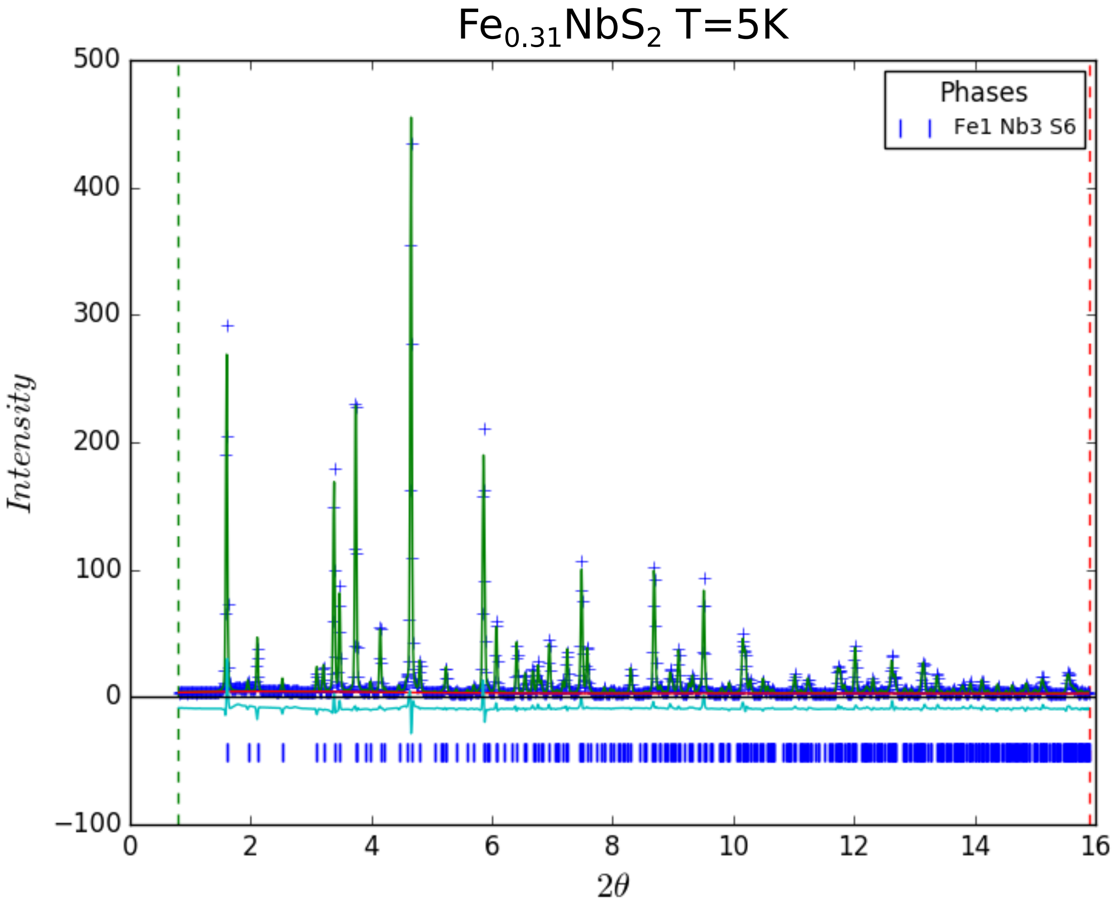

FIG. S4. Rietveld refinement of high-resolution synchrotron powder XRD measurements on $Fe_{0.31}NbS_2$ at $5K$. Calculated lattice parameters are a= 5.654858 +/- 0.000182 Å, c=11.935061 +/- 0.000401 Å. The cross markers are data with the fit shown by the green curve, and the difference between the fit and data is shown in cyan. Vertical lines denote structural peak positions.

two domain orientations, while the domain with principal axis perpendicular to the pulse is favored following a vertical current pulse. In a zigzag-dominated sample as well, current pulses favor domains whose principal axes are not parallel to the pulse. The domain configurations in panels (c) and (e) have opposite conductivity anisotropies, as do those in panels (d) and (f) [1], so that (g) when stripe and zig-zag orders coexist, there will be competing switching responses, as shown schematically in (h). Note the similarity between the black curve in (h) and the observed signal in Fig. 4, with a small intitial response with an opposite sign flip to the main response, and a decreasing response after an initial peak.

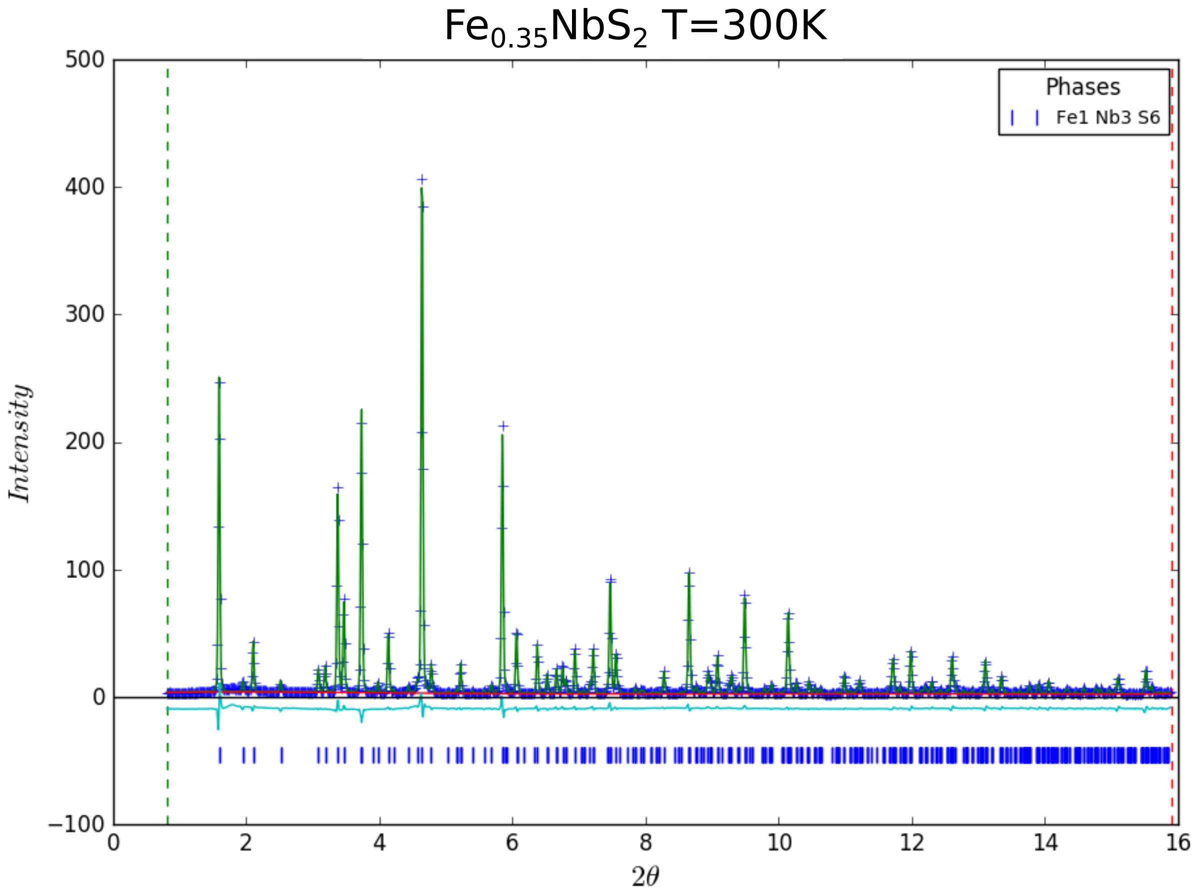

FIG. S5. Rietveld refinement of high-resolution synchrotron powder XRD measurements on $Fe_{0.35}NbS_2$ at $300K$. Calculated lattice parameters are a=5.660797 +/- 0.000115 Å, c = 11.994610 +/-0.000260 Å. The cross markers are data with the fit shown by the green curve, and the difference between the fit and data is shown in cyan. Vertical lines denote structural peak positions.

More devices

There is some variation in switching response between devices, as their dimensions, exact concentrations, geometries, and mounting conditions vary slightly. See Figs. S14 and S15 for examples.

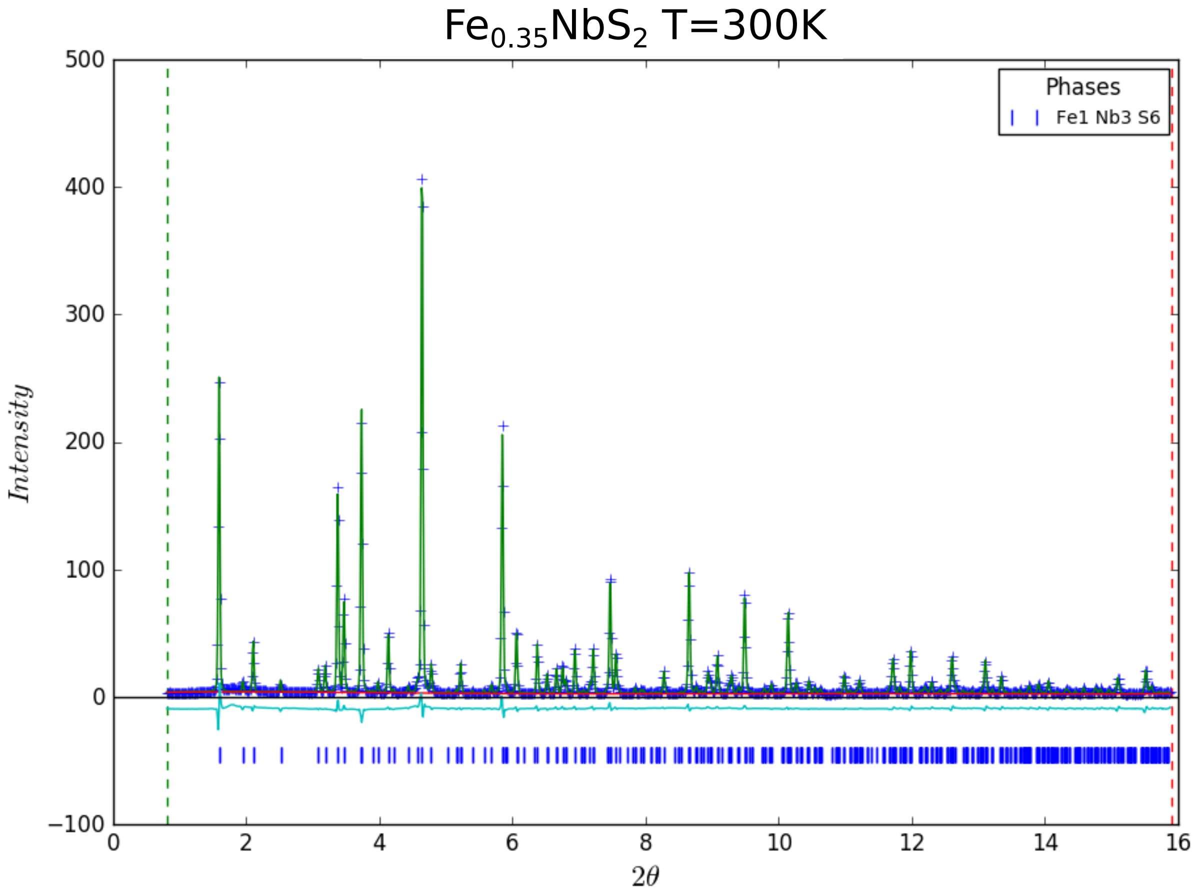

FIG. S6. Rietveld refinement of high-resolution synchrotron powder XRD measurements on $Fe_{0.35}NbS_2$ at $10K$. Calculated lattice parameters are a=5.654070 +/- 0.000153 Å, c = 11.968898 +/-0.000296 Å. The cross markers are data with the fit shown by the green curve, and the difference between the fit and data is shown in cyan. Vertical lines denote structural peak positions.

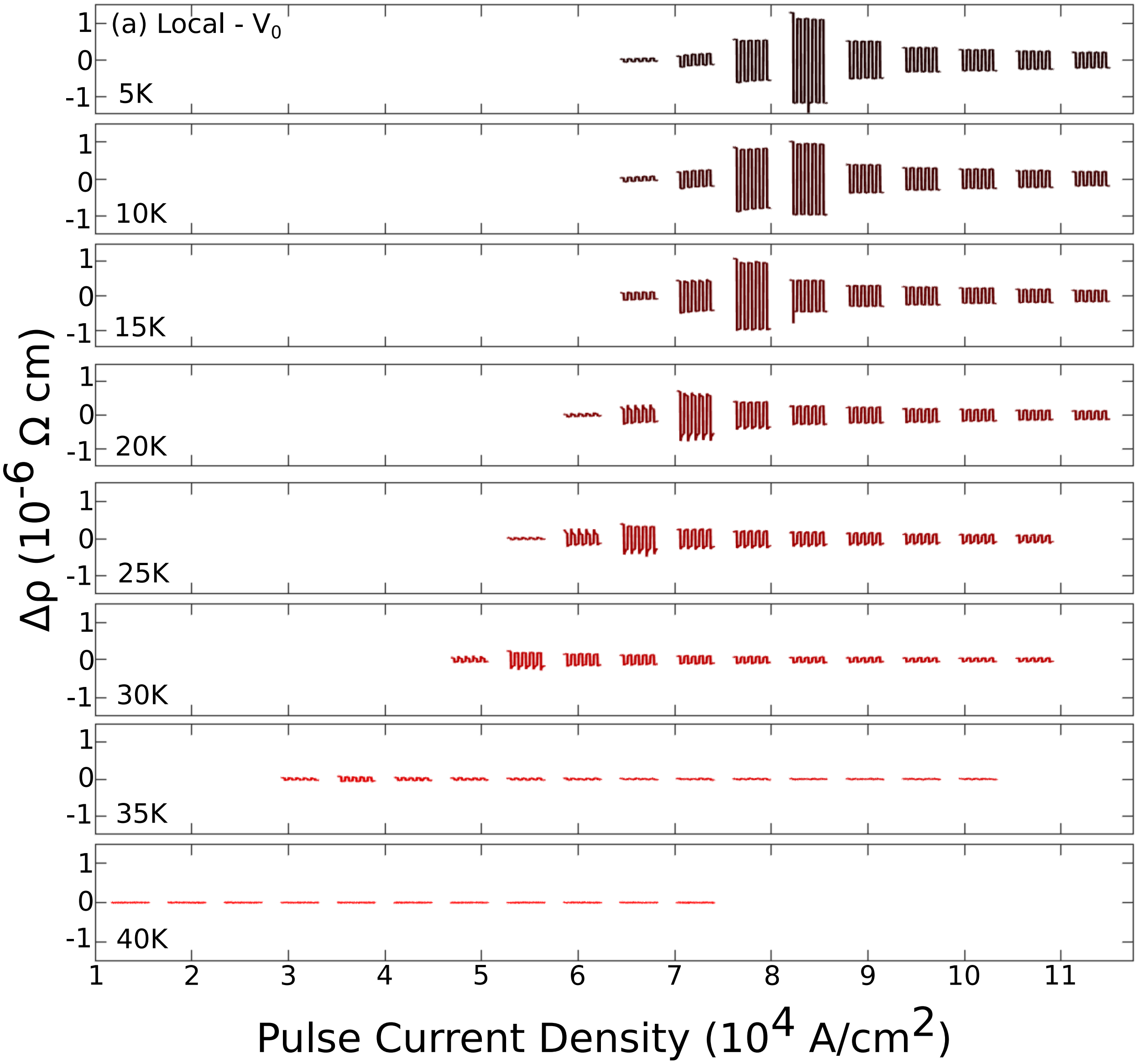

FIG. S7. Local switching measurement as a function of pulse current density, shown at temperatures from $5K$ to $40K$.

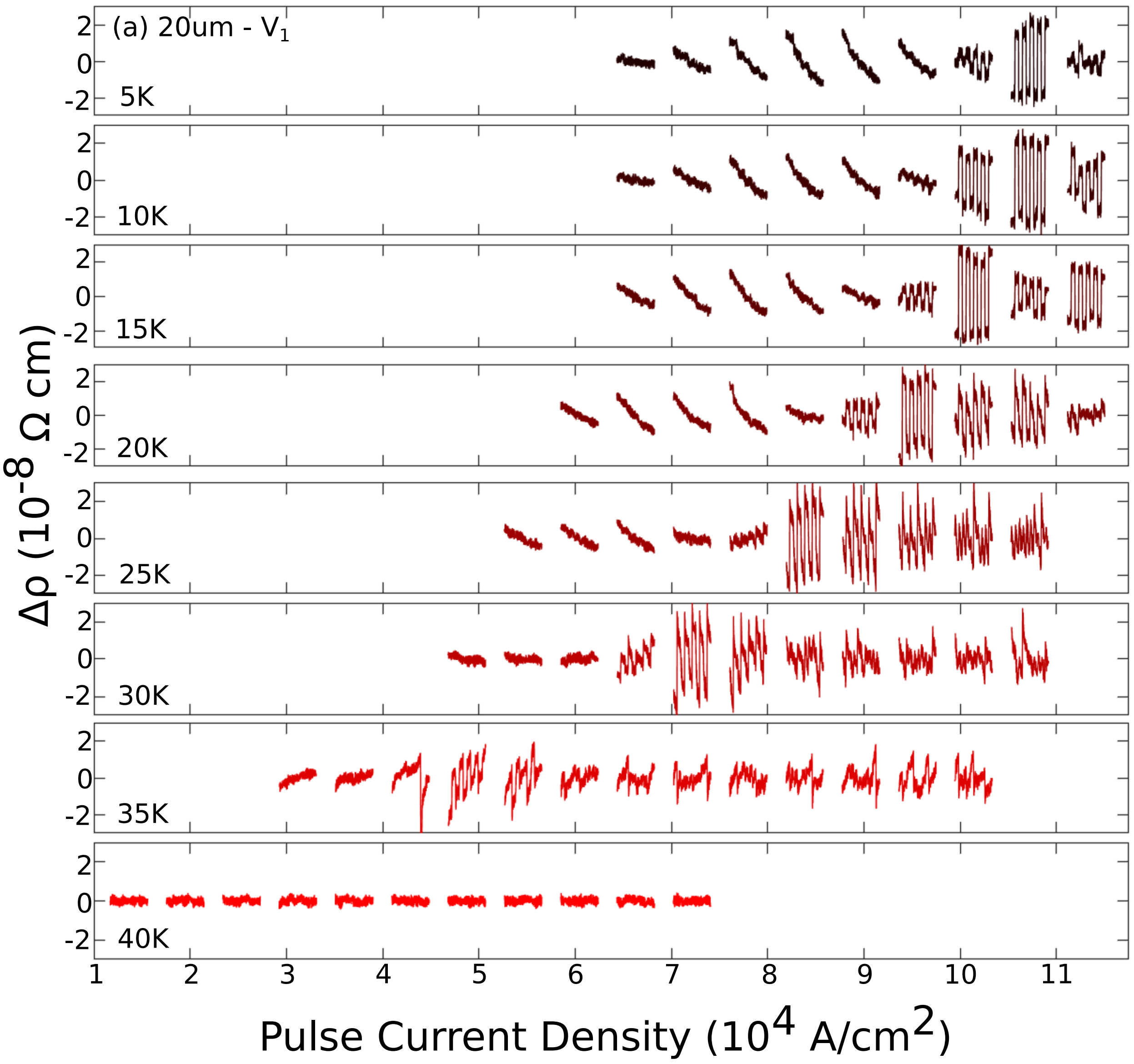

FIG. S8. non-local ($20\mu m$ from the center of the device) switching measurement as a function of pulse current density, shown at temperatures from $5K$ to $40K$.

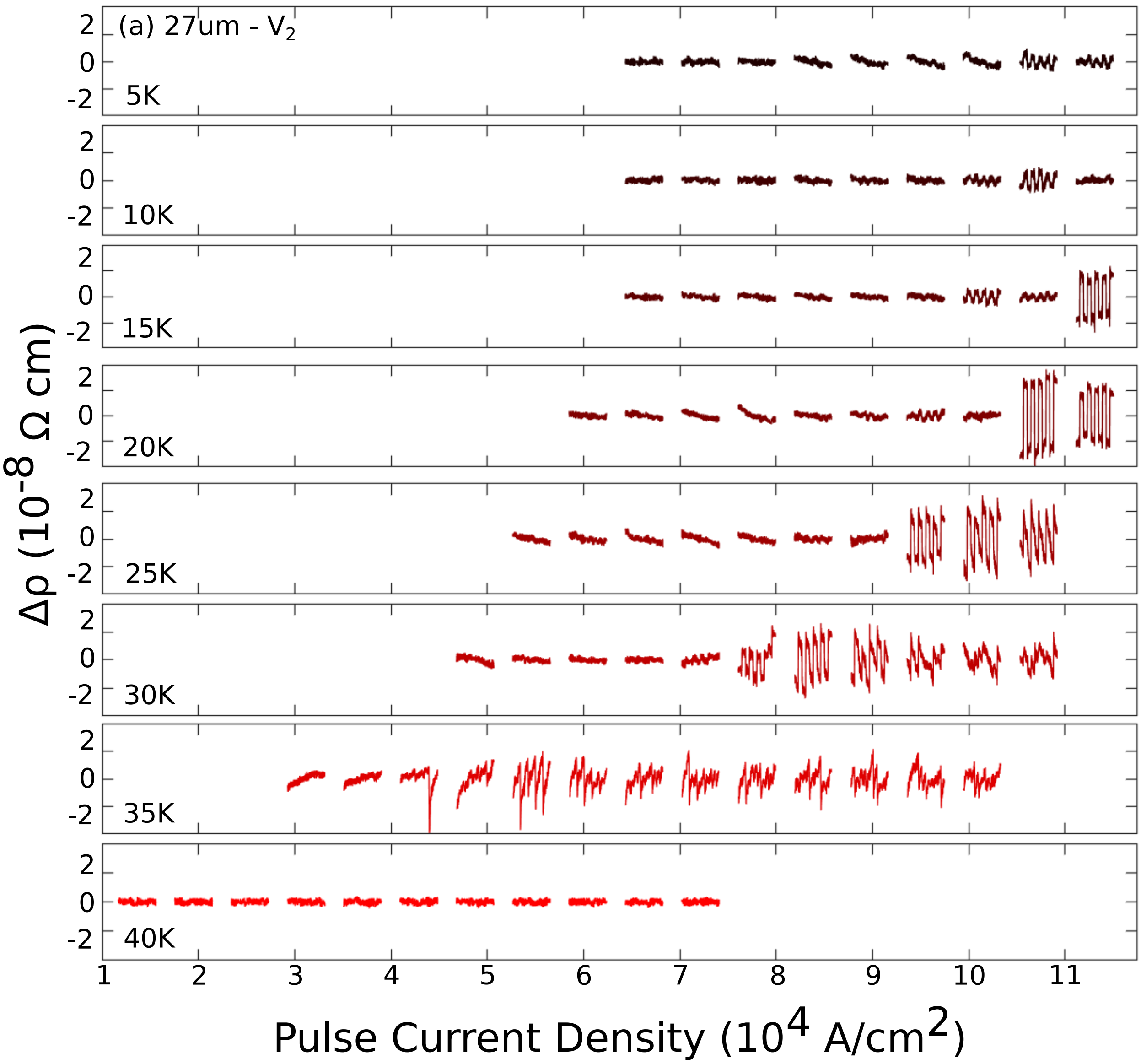

FIG. S9. non-local ($27\mu m$ from the center of the device) switching measurement as a function of pulse current density, shown at temperatures from $5K$ to $40K$.

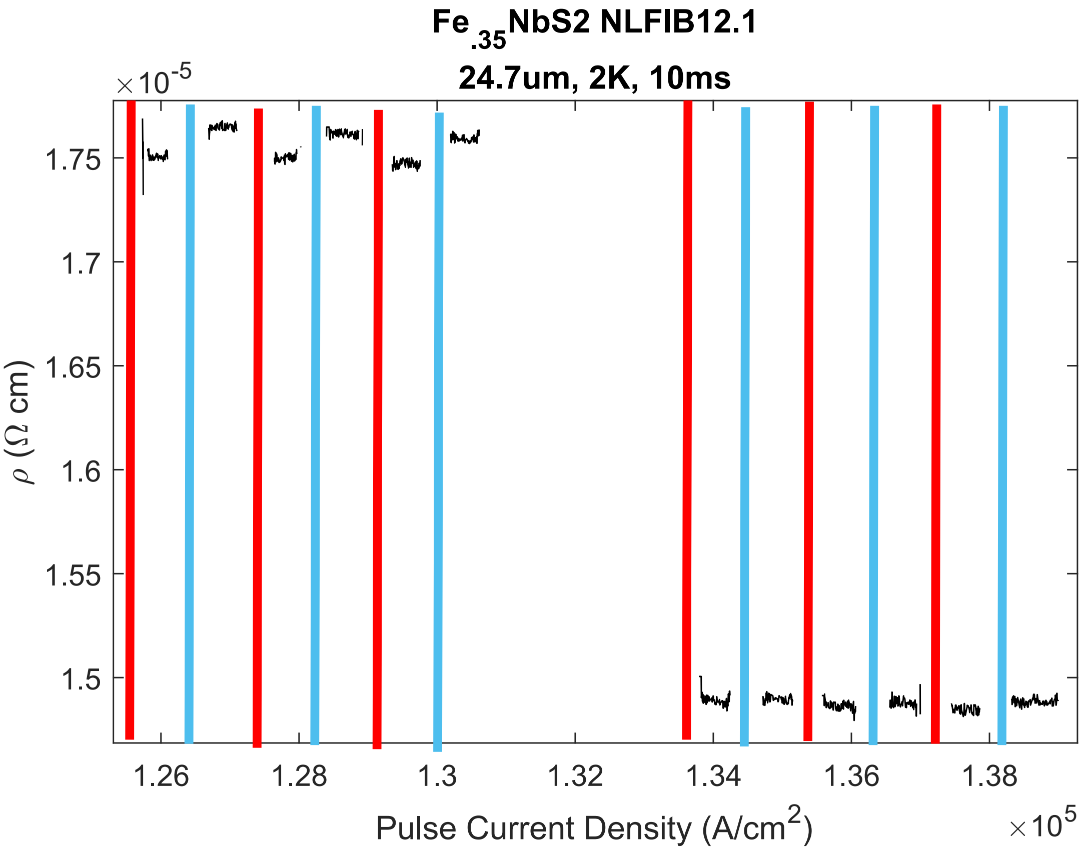

FIG. S10. Switching measured 25μm from the center of a device at a current density that results in switching (left) and that does not result in switching (right), with the AC probe current turned off and its leads detatched during the switching events themselves. Vertical lines indicate switching events, and readouts from the lockins while the leads were detached have been omitted.

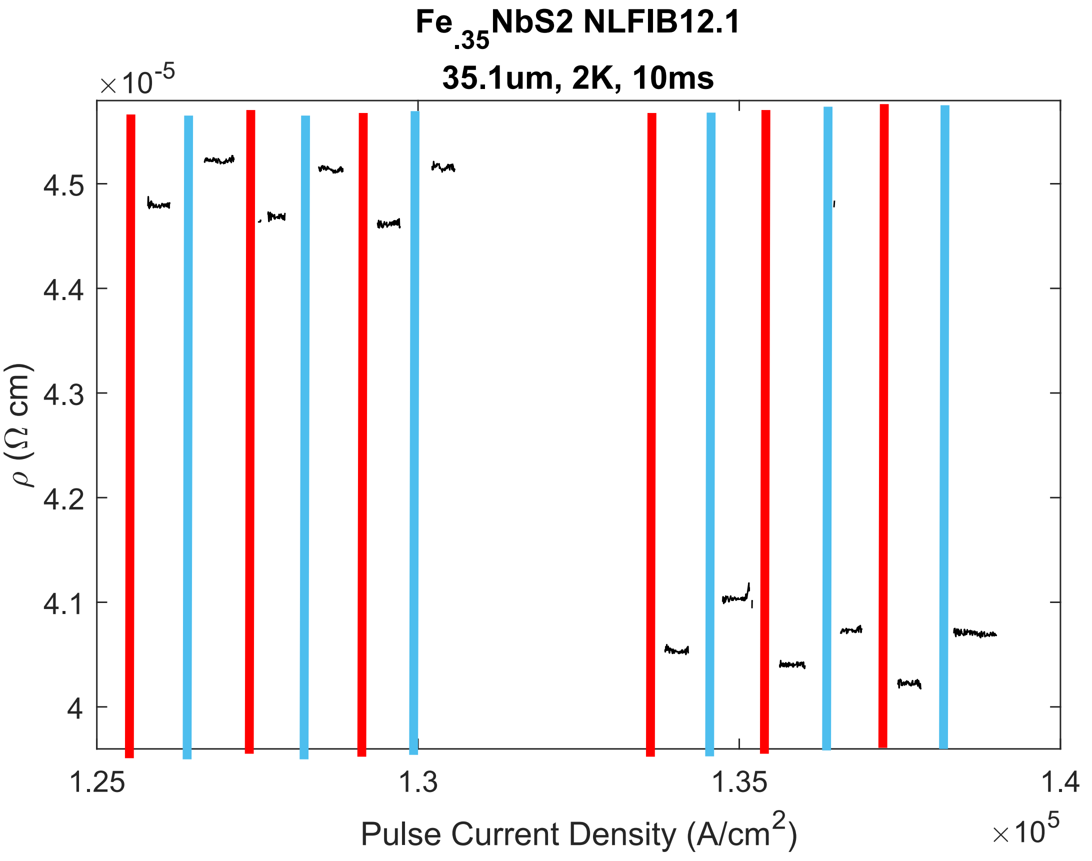

FIG. S11. Switching measured $35\mu m$ from the center of a device at two different current densities that result in switching (left and right), with the AC probe current turned off and its leads detatched during the switching events themselves. Vertical lines indicate switching events, and readouts from the lockins while the leads were detached have been omitted.

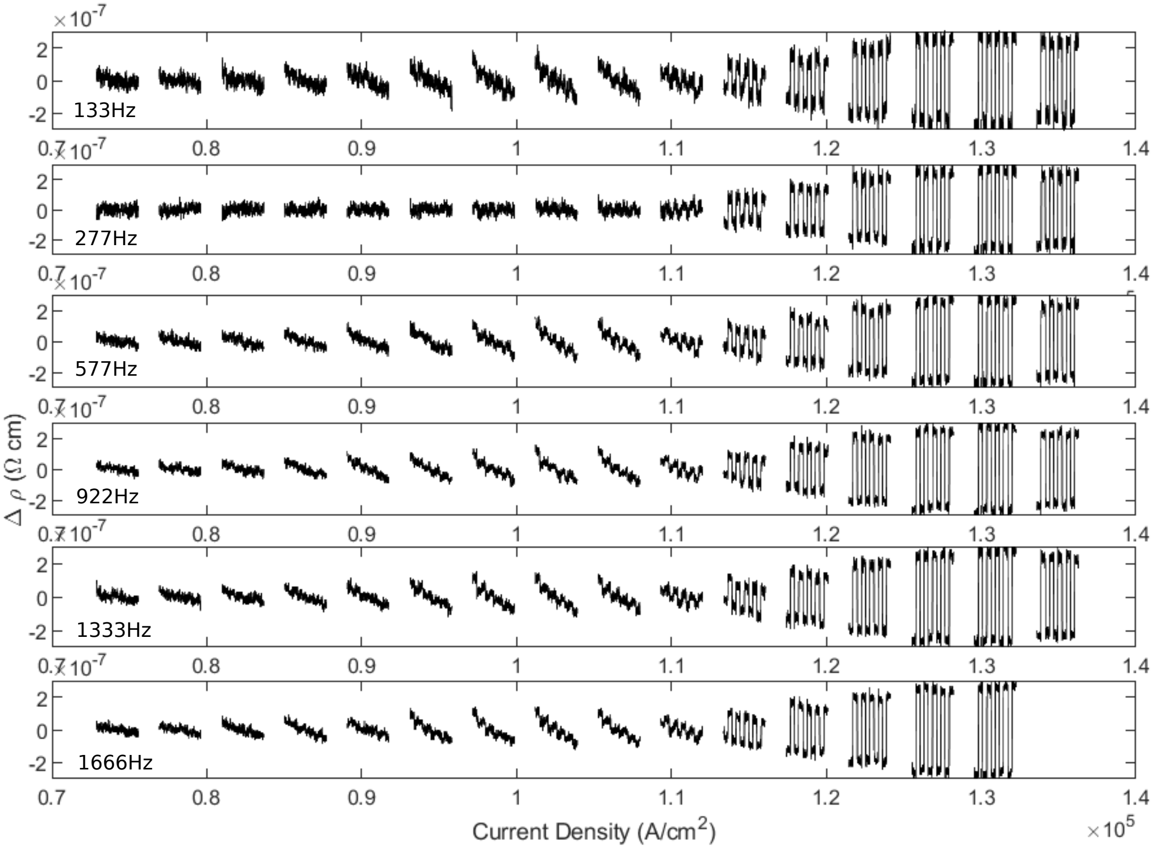

FIG. S12. Switching measured $35\mu m$ from the center of a device at a current density that result in switching, with AC probe frequencies ranging from $133Hz$ to $1666Hz$. The noise changes from measurement to measurement, but the switching behavior notably does not.

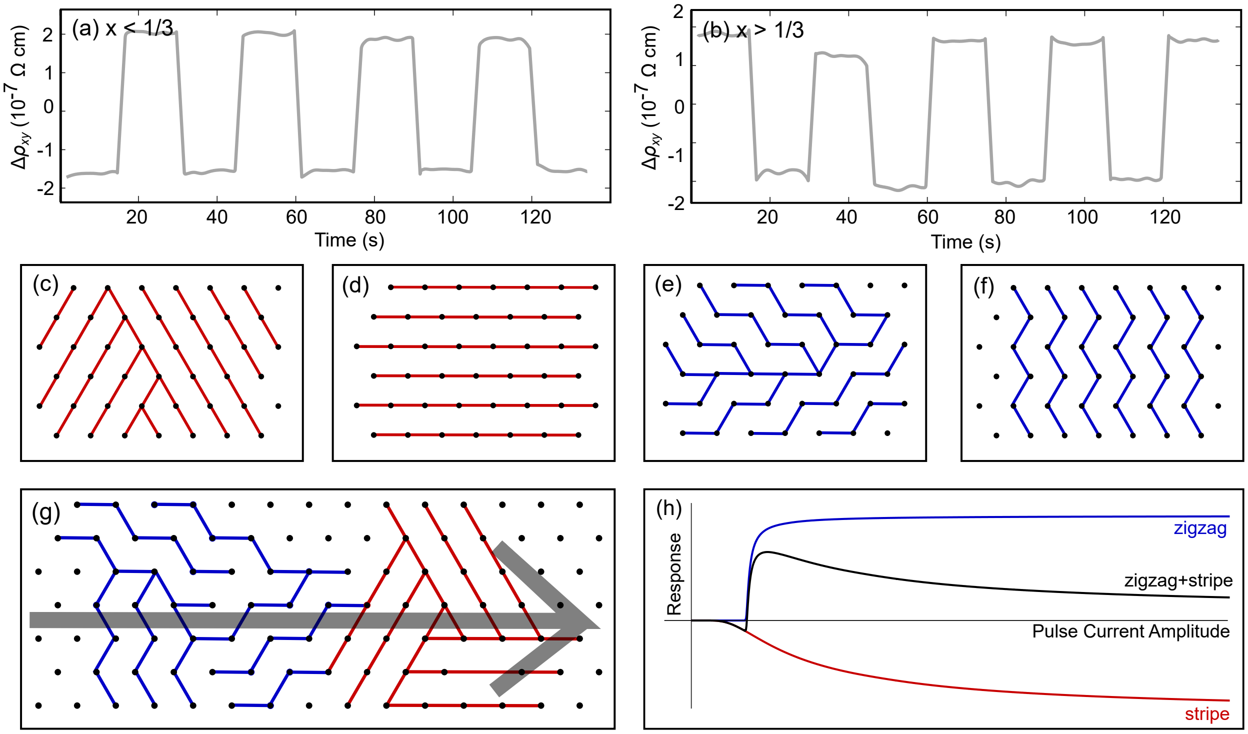

FIG. S13. (a) Transverse resistance switching response in Fe_xNbS_2 with $x < 1/3$. Data were taken at $2K$, with pulse current amplitudes of approximately $15 \times 10^4 A/cm^2$, in the regime where switching has moved beyond the initial anomalous region as seen around $8.5 \times 10^4 A/cm^2$ in Fig.**??**A. In both cases, the first pulse and then every other subsequent pulse was normal to a crystal facet. (b) Transverse resistance switching response in Fe_xNbS_2 with $x > 1/3$. With identical device geometries, a pulse which brought $x < 1/3$ to a low resistance state brings $x > 1/3$ to a high resistance state, and vice versa. (c-f) Illustration of stripe and zigzag domains. Circles are iron atoms in one plane. Lines drawn between iron atoms indicate their spins are aligned. (c) Domain configuration preferred following a horizontal pulse in a stripe-dominated sample. (d) Domain configuration preferred following a vertical pulse in a stripe-dominated sample. (e) Domain configuration preferred following horizontal pulse in a zigzag-dominated sample. (f) Domain ocnfiguration preferred following vertical pulse in a zigzag-dominated sample. (h) Proposed combination of zigzag and stripe responses in zigzag-dominated sample. Note the similarity between the black curve and the observed signal in Fig. 4, with a small intitial response with an opposite sign flip to the main response, and a decreasing response after an initial peak.

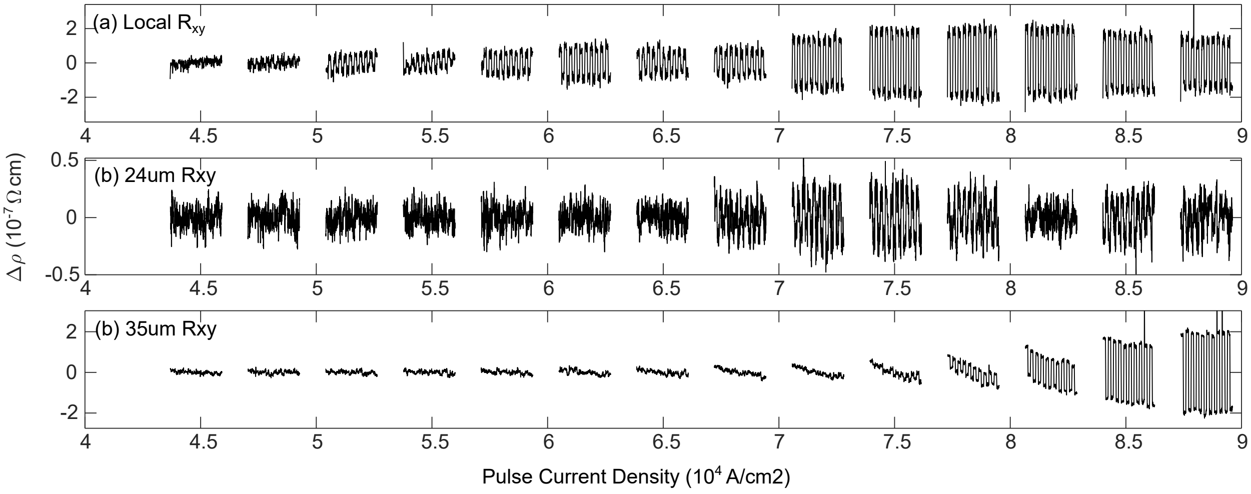

FIG. S14. Switching response in a device with a different current density dependence than those shown in the main text. (a) Local response is nonmonotonic, showing a change in sign as a function of pulse current density. (b) $24\mu m$ from the center of the device, the non-local response is small but shows a change in sign as well. The first switching responses have an opposite sign to the first local switching responses. (c) $35\mu m$ from the center of the device, the non-local response is comparable to the maximum local response. Compared to the initial onset of switching in the local portion of the device, the response seen here is larger and has an opposite sign.

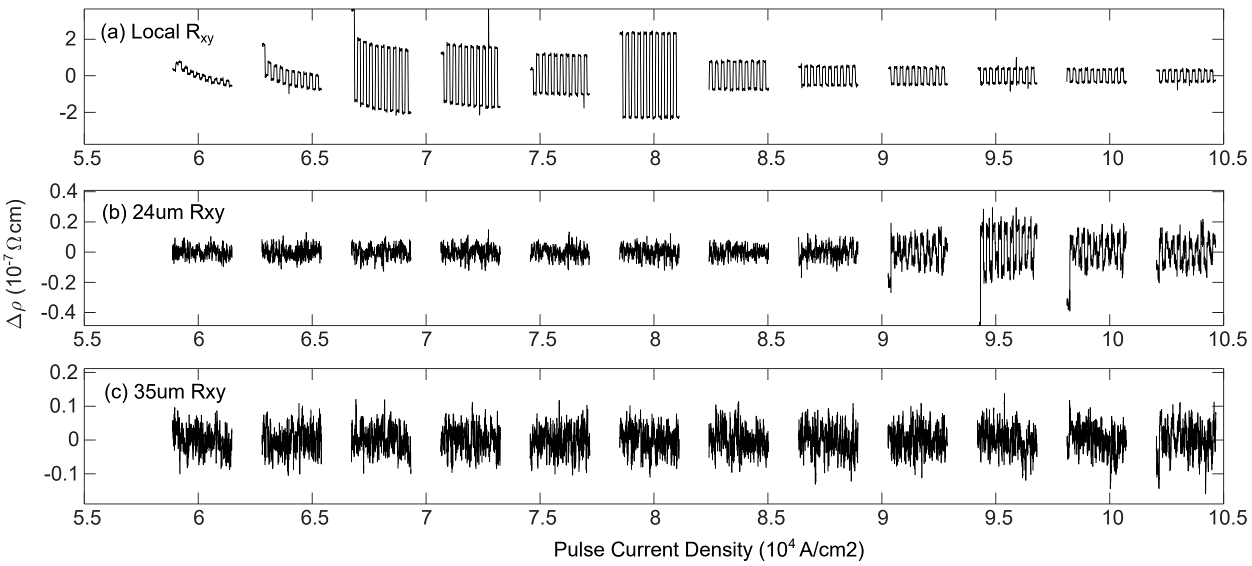

FIG. S15. Switching response in a device with a different current density dependence than those shown in the main text. (a) Local response is nonmonotonic, showing two peaks followed by the decreasing amplitude characteristic of most devices. The presence of two peaks is suggestive of inhomogeneous iron content or a twist in the stack of layers. (b) $24\mu m$ from the center of the device, the non-local response is small but has a sign opposite that of the local response. (c) $35\mu m$ from the center of the device, a non-local response is not observed, presumably because the measurement did not extend to high enough current densities.

REFERENCES

[1] S. F. Weber and J. B. Neaton, Phys. Rev. B **103**, 214439 (2021).
